# Conserved intergenic sequences revealed by CTAG-profiling in *Salmonella*: thermodynamic modeling for function prediction

**DOI:** 10.1038/srep43565

**Published:** 2017-03-06

**Authors:** Le Tang, Songling Zhu, Emilio Mastriani, Xin Fang, Yu-Jie Zhou, Yong-Guo Li, Randal N. Johnston, Zheng Guo, Gui-Rong Liu, Shu-Lin Liu

**Affiliations:** 1 Systemomics Center, College of Pharmacy, and Genomics Research Center (State-Province Key Laboratories of Biomedicine-Pharmaceutics of China), Harbin Medical University, Harbin, China; 2 HMU-UCFM Centre for Infection and Genomics, Harbin Medical University, Harbin, China; 3 Department of Ecosystems and Public Health, University of Calgary, Calgary, Canada; 4 Department of Infectious Diseases of First Affiliated Hospital, Harbin Medical University, Harbin, China; 5 Department of Biochemistry and Molecular Biology, University of Calgary, Calgary, Canada; 6 College of Bioinformatics Science and Technology, Harbin Medical University, Harbin, China; 7 Department of Microbiology, Immunology and Infectious Diseases, University of Calgary, Calgary, Canada

## Abstract

Highly conserved short sequences help identify functional genomic regions and facilitate genomic annotation. We used *Salmonella* as the model to search the genome for evolutionarily conserved regions and focused on the tetranucleotide sequence CTAG for its potentially important functions. In *Salmonella*, CTAG is highly conserved across the lineages and large numbers of CTAG-containing short sequences fall in intergenic regions, strongly indicating their biological importance. Computer modeling demonstrated stable stem-loop structures in some of the CTAG-containing intergenic regions, and substitution of a nucleotide of the CTAG sequence would radically rearrange the free energy and disrupt the structure. The postulated degeneration of CTAG takes distinct patterns among *Salmonella* lineages and provides novel information about genomic divergence and evolution of these bacterial pathogens. Comparison of the vertically and horizontally transmitted genomic segments showed different CTAG distribution landscapes, with the genome amelioration process to remove CTAG taking place inward from both terminals of the horizontally acquired segment.

Bacterial genomes may contain highly conserved short sequences that are lineage-specific, such as the tetranucleotide sequence CTAG in *Escherichia coli* and *Salmonella*^1^,^2^. Genomic regions containing such conserved short sequences carry information about the evolution and phylogenetic divergence of the bacteria, but so far such information, especially that embedded in the intergenic regions, has not been efficiently extracted, due largely to the lack of theoretical or methodological strategies to reveal such conserved sequences. As a result, intergenic regions of the bacterial genomes have been poorly annotated in contrast to genes, which researchers often compare with those of *E. coli*^3^ for annotation, taking the advantage that functions of many of the *E. coli* genes have been validated by molecular biology experiments. Such a situation calls for novel approaches to facilitate genomic annotation, and characterization of conserved short sequences may prove instrumental in the identification of functional intergenic sequences.

For this purpose, we have focused on the identification and characterization of short sequences in the bacterial genome, using representative *Salmonella* lineages as the models. Previously, we reported that the CTAG-containing endonuclease cleavage sites such as that of XbaI (TCTAGA) are highly conserved^4^,^5^. In a recent study, we found that many of such cleavage sites are located in common intergenic regions across *Salmonella* and *E. coli*^1^. This finding strongly suggests the potential importance of such sequences and brings up the hopes that the identification and functional analyses of such genomic regions may facilitate bacterial genome annotation and functional studies. We anticipate that the highly conserved intergenic regions may be involved in gene expression regulation and pathogenic evolution. In the case of *Salmonella*, more enhanced genome annotation strategies are especially needed for understanding the divergence processes that have created diverse and distinct pathogens from common ancestors.

One of the main reasons for us to use *Salmonella* as the models in this study is that this genus contains the most widely distributed bacterial lineages known to date, which are genetically highly similar among them but pathogenically each drastically distinct^6^, with the genetic factors that have made these closely related bacteria different pathogens remaining largely unknown. Since the first isolation of a *Salmonella* strain from a typhoid patient in 1881, more than 2600 *Salmonella* lineages, classified as serotypes based on their different combinations of the somatic (O) and flagellar (H) antigens, have been documented^7^. The genetic similarity among the *Salmonella* serotypes was revealed by comparison of genome structures in the last century^8^,^9^ and genomic sequences since the beginning of this century^10^,^11^,^12^, although these bacteria cause different diseases, from self-limited gastroenteritis (such as *S. typhimurium, S. enteritidis*, etc.) to potentially fatal systemic infections like typhoid (such as *S. typhi*)^13^. The current bacterial taxonomy tends to classify the gastroenteritis-causing *S. typhimurium* and the typhoid-causing *S. typhi* into the same subspecies as *Salmonella enterica* subspecies *enterica* serovars Typhimurium and Typhi, respectively, however we and many other authors have continued using the traditional nomenclature to avoid confusion; we explained the rightfulness of treating *S. typhimurium* and *S. typhi* as separate species in a recent perspective article^14^.

Previous studies have shown that the *Salmonella* lineages that elicit similar disease phenotypes may have evolved by either divergent or convergent processes^12^. However, whether similar pathogens may use the same set or overlapping sets of genes for the infections remains unclear. Further, very possibly, some elements encoded by intergenic sequences might be involved in the modulation of virulence expression. Complicating this issue is the fact that more than half of the genes in *Salmonella* genomes still await convincing annotation, and deciphering the seemingly non-coding sequences in the intergenic regions is even more challenging.

In this study, we profiled the CTAG sequences, which are relatively rare in *Escherichia coli* and *Salmonella*^15^, in representative *Salmonella* serotypes. To evaluate their level of conservation in evolution, we determined their genomic distribution in comparison with *E. coli* and other bacteria. We hypothesize that it is the potential biological significance of the intergenic CTAG-containing sequences that have made them highly conserved, therefore they survived the elimination process by the Very Short Patch (VSP) repair mechanism^2^. We ranked the profiled CTAG sequences according to their theoretical importance in function by their levels of evolutionary conservation. As the most highly conserved sequences may retain key functions, we focused on the CTAG sequences that are present in the least annotated intergenic regions across *Salmonella* and *E. coli*. Computer modeling demonstrated that, for the CTAG sequences conserved in bacteria across the Enterobacteriaceae genera, substitution of any of the four nucleotides may disrupt the stem-loop structure of the CTAG-containing intergenic sequence, potentially affecting their biological functions. We also documented CTAG sequence degeneration patterns and found that the CTAG sequence decays in serotype-specific ways. Of particular interest, the degeneration processes of CTAG seemed to be current and still going on at different stages in the genome.

## Methods

### Bacterial strains

Information on all bacterial strains used in this study can be found at the *Salmonella* Genetic Stock Center (http://www.ucalgary.ca/~kesander/), and the accession numbers of the sequenced genomes can be found at http://www.ncbi.nlm.nih.gov/genome. We use the traditional *Salmonella* nomenclature for reasons detailed in a previous publication^14^.

### Construction of phylogenetic trees

We aligned the 16S rDNA sequences of the bacterial strains in comparison using the Clustal X program and constructed the phylogenetic trees by MEGA6 using the Neighbor-Joining algorithm.

### General strategies of computer modeling to predict secondary structures of short sequences

**Figure 1:**
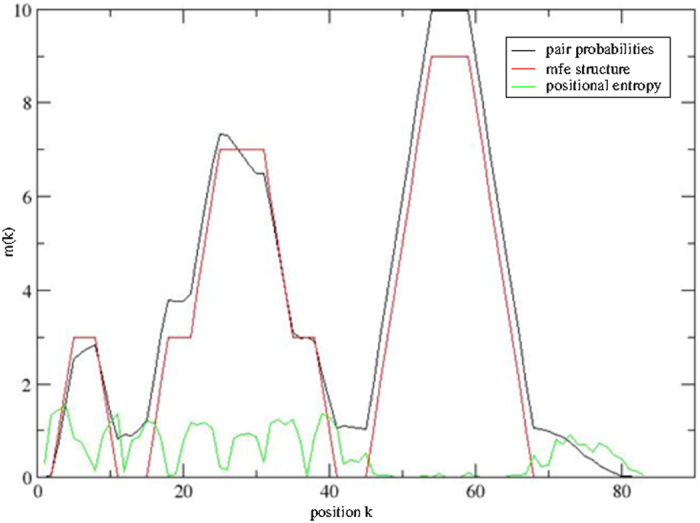
Mountain plot representing the modeled secondary structure by height versus position. The height m(k) is given by the number of base pairs enclosed at position k. Three curves are shown: the MFE structure (red), the pairing probabilities (black) and a positional entropy curve (green). Well-defined regions are identified by low entropy.

The modeling was conducted based on the Free Energy Minimization method^16^,^17^,^18^. As free energy models typically assume that the total free energy of a given secondary structure for a molecule is the sum of independent contributions of adjacent or stacked base pairs in stems, we particularly focused on the stem-loop structures. Specifically, we employed the Vienna RNA Package (http://www.tbi.univie.ac.at/RNA/), which consists of a C code library and several stand-alone programs, including RNAfold. This program reads RNA sequences from standard inputstdin, calculates their minimum free energy (mfe) structure and prints to standard output. The *mountain.pl* script produces a mountain plot, which is an xy-diagram plotting the number of base pairs enclosing a sequence position. The resulting plot shows three curves (red, black and green, respectively; Fig. 1), with the red one showing two peaks derived from the MFE structure, the black one demonstrating the pairing probabilities, and the green one indicating the positional entropy. Well-defined regions are identified by low entropy. By superimposing several mountain plots, the structures can easily be compared.

To test the reliability of the modeling, we also used the Maximum Expected Accuracy method by the program CONTRAfold^19^ and predicted the most probable structure and the pseudo-knot-free structures by maximizing the sum of the base-paired and single-stranded nucleotide probabilities, called expected accuracy, where pairing probabilities can be weighted by a specific factor. CONTRAfold uses probabilistic parameters learned from a set of RNA secondary structures to predict base-pair probabilities and then predicts structures using the maximum P (i, j) expected accuracy approach. In this study, we referred to The MaxExpect program (http://rna.urmc.rochester.edu/RNAstructureWeb), which is part of the RNAstructure package by Mathews Lab, University of Rochester Medical Center, Department of Biochemistry and Biophysics. To run the programs for the prediction, we used an HPC Cluster based on Ubuntu 14.04.2 LTS (Trusty), employing the Sun Grid Engine 6.2u5–7.3 amd64 as queue manager and scheduler to accept jobs. We ran the programs on the cluster in *“trivial parallel computing”* way and obtained the results from RNAfold v. 2.19 and MaxExpect v. 5.6 linked to Perl v.5.18.2. Throughout the modeling, we used both strategies to crosscheck each other’s performance and evaluate the quality of the obtained predictions.

### Using the RNAfold program to model the structure

We used two scripts, *mountain.pl* and *relplot.pl*, We used two scripts, mountain.pl and relplot.pl, which are part of the core routines on-board with the Vienna RNA Package (www.tbi.univie.ac.at/RNA/), with the former for predicting pair probabilities within the equilibrium ensemble and the latter for producing a diagram of the predicted structure containing also information about probability. The Perl script *relplot.pl* adds reliability information to a RNA secondary structure plot and computes a well-definedness measure, which we call “positional entropy” (Fig. 1).

### Secondary structure prediction by the MEA approach

Using the MaxExpect program, we executed the following command: MaxExpect –sequence LT2-SpeI.fasta LT2-SpeI.out –gamma 1 –percent 10 –structures 20 –window 3″. This will generate a file name containing information about the predicted structure.

### Illustration of CTAG frequency and landscape on genomic sequences

The frequency of the CTAG sequence was analyzed using our own Perl scripts. To profile the CTAG sequences, we first looked for the positions of all CTAG and then extended the analysis to 50 bp of the genomic sequence both up- and down-stream of the CTAG. We used this 104 bp sequence as query to search in BLAST db (like subject sequence). To illustrate the CTAG frequency on the genomic sequence, we showed the numbers of the tetranucleotide per 10 kb window.

### Determining the level of sequence conservation

To determine how a given sequence is conserved across different bacteria, we searched it against the NCBI databases by the BLAST service and ranked the level of conservation by the set of parameters including Max and Total scores, E-values and sequence coverage and percent identity. In the case of the inter-*lpp-pykF* sequence, we used the sequence as query in the search against the database (https://www.ncbi.nlm.nih.gov/genome) that contains all published genomes of Enterobacteriaceae family.

## Results

### Profiling the CTAG sequence in bacterial genomes

The tetranucleotide sequence CTAG is remarkably under-abundant in *E. coli* and *Salmonella* as previously evidenced by the relatively small numbers of endonuclease cleavage sites containing CTAG such as XbaI (TCTAGA), BlnI or AvrII (CCTAGG), and SpeI (ACTAGT)^5^,^15^,^20^, a phenomenon of biased codon usage intensively studied in *E. coli*^21^,^22^. *E. coli* and closely related bacteria contain the Very Short Patch repair system, which tends to eliminate CTAG where possible in the genome^2^,^23^. To quantitatively validate the scarcity of the CTAG sequence in *Salmonella* and *E. coli*, we profiled the tetranucleotides consisting of one each of C, T, A and G ordered randomly in the genome by the formula of:


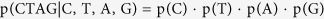


In *S. typhimurium* LT2, p(C), p(T), p(A) and p(G) are 0.26, 0.24, 0.24 and 0.26^10^,^22^, respectively, and the frequency of a random combination of C, T, A and G comes to 0.00389376, which would lead to an estimated number of a random combination of C, T, A and G to be 18914 in the genome of *S. typhimurium* LT2, which is 4857432 bp. When we actually profiled the tetranucleotide sequences consisting of one each of C, T, A and G in *S. typhimurium* LT2 and representative strains of *S. typhi, S. paratyphi* A, B, C, and *S. gallinarum* in comparison with *E. coli* K12, we found that the majority of the combinations have numbers greater than twelve thousand in all *Salmonella* genomes analyzed and the numbers of many of them are close to the estimated 18914 (Table 1), e.g., CAGT (18347), TGAC (19229), ACTG (18472) or GATC (19168).

**Figure 2:**
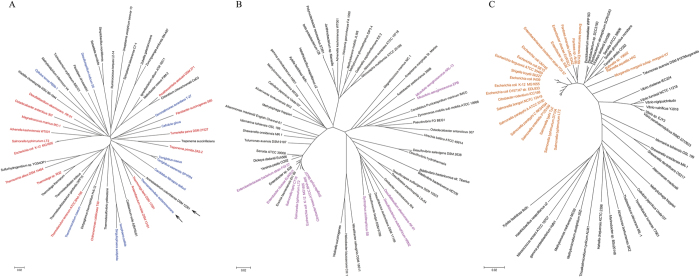
Phylogenetic tree of bacterial strains based on 16S rDNA sequence comparison. (**A**) Bacterial strains representing a wide range of phyla; color categories for GC percentages: black, GC up to 45%; red, GC 46–55%; blue, GC >55% (see Supplementary Table 1). (**B**) Bacterial strains representing main branches of the Proteobacteria Phylum; purple color indicates bacteria that had lowest CTAG frequencies among the strains compared (see Supplementary Table 2). (**C**) Bacterial strains representing main branches of the Gammaproteobacteria Class; orange color indicates bacteria that had lowest CTAG frequencies among the strains compared (see Supplementary Table 3).

To test the postulation that the CTAG sequence scarcity seen in *Salmonella*^1^ is not a general feature of bacteria at large, we screened sequenced strains of phylogenetically diverse bacteria to document the number of the CTAG sequence and determine its frequency per kb genome (Supplementary Table 1). We found that the CTAG sequence frequency varies widely among the bacteria, from as low as 0.023 per kb in *Thermodesulfovibrio yellowstonii* DSM 11347 (Phylum: Nitrospira, Class: Nitrospira, Order: Nitrospirales, Family: Nitrospiraceae; Chromosome 1, GC percentage: 34%) to as high as 3.933 per kb in *Thermobaculum terrenum* ATCC BAA-798 (Thermobaculum; GC percentage: 48%). *E. coli* K12 and *S. typhimurium* LT2 had a CTAG sequence frequency of 0.191 and 0.175 per kb, respectively. It is worth noting that bacteria with CTAG sequence frequencies lower than those of *E. coli* and *Salmonella* were seen in both low and high GC categories, demonstrating that the CTAG sequence frequency is not correlated with GC contents. Even within a narrow range of GC compositions, such as GC percentages of >45 and <=55%, which cover those of *E. coli* and *Salmonella* (around 51–52%), CTAG sequence frequencies vary remarkably, e.g., from 0.095 per kb in *Prosthecochloris aestuarii* DSM 271 (GC 50%) to 3.933 per kb in *T. terrenum* ATCC BAA-798 (GC 48%). Additionally, at the level of phyla, bacteria having very different GC contents and CTAG sequence frequencies are mixed without a noticeable phylogenetic tendency. For example, bacteria as closely related as *Aminobacterium colombiense* DSM 12261 and *Thermanaerovibrio acidaminovorans* DSM 6589 may have GC compositions as different as 45% and 64%, and CTAG sequence frequencies as different as as 0.988 and 0.684 per kb, respectively (see arrows in Fig. 2A, and Supplementary Table 1). Therefore, at the highest evolutionary branches, bacteria do not exhibit phylogenetic tendencies of CTAG frequency or GC content.

Within the Proteobacteria Phylum, bacteria among different Classes have a much narrower range of GC compositions, i.e., from 45 to 55%, but their CTAG sequence frequencies still vary broadly, from 0.125 to 4.590 per kb (Supplementary Table 2), demonstrating that low CTAG sequence frequencies are not common either within the Proteobacteria branch. Of interest, bacteria with lower CTAG frequencies tended to cluster to the Gammaproteobacteria Class, which contains the Enterobacteriaceae including the Genera *Salmonella* and *Escherichia* (Fig. 2B). When we focused on representative bacteria among the Gammaproteobacteria branches, we found that bacteria with low CTAG frequencies, such as *Salmonella* and *Escherichia*, where the CTAG frequencies are around 0.2 per kb or lower, mostly belong to the Enterobacteriaceae Family (Fig. 2C), although many Enterobacteriaceae bacteria had much higher CTAG frequencies, e.g., >0.7 per kb in *Yersinia* (Supplementary Table 3). Therefore, CTAG frequency as low as those of *Salmonella* and *Escherichia* is not a general feature of bacteria of the Enterobacteriaceae Family but is common only to *Salmonella, Escherichia* and their close relatives (Table 2).

### Evolutionary implications of the CTAG sequence

Assuming that the common ancestor of *Salmonella* and *E. coli* had a much higher frequency of CTAG 200 million years back in evolution, one would anticipate seeing different patterns of the degeneration process of the CTAG sequence among the descendants of the assumed ancestor. In *Salmonella*, all serotypes have diverged from their ancestors during adaptation to their specific niches under distinct selection pressures. To look into the hypothesized degeneration patterns of the CTAG sequence in different lineages of the bacteria, we profiled CTAG and those deemed homologous to CTAG but with one or two of the four nucleotides replaced by other nucleotides in representative *Salmonella* strains and found lineage-specific patterns of the degeneration. For example, the CTAG in gene *yiaE* is conserved in *S. typhimurium* and *S. heidelberg* but is degenerated to CTGG in the other *Salmonella* lineages compared; similarly, the CTAG between genes *yjjY* and *lasT* is conserved in most analyzed *Salmonella* lineages except *S. heidelberg*, where this tetranucleotide is degenerated to ATAG (Table 3). Overall, each analyzed *Salmonella* lineage has a distinct pattern of CTAG degeneration, reflected by “signature CTAG degeneracies” such as the ATAG between genes *yjjY* and *lasT* in *S. heidelberg* or combinations of specific CTAG degeneracies, which are present in all strains of a given *Salmonella* lineage (lineage-specific CTAG degeneration pattern; Supplementary Table 4). For instance, all eight strains of *S. typhimurium* analyzed in this study have a common pattern and all three strains of *S. typhi* have another pattern (e.g., the sequence CTAG at the genomic location 2948212–2948215 of *S. typhimurium* LT2 and all other analyzed *Salmonella* strains is conserved except *S. typhi*, in which the sequence is CTAT; Supplementary Table 4). Interestingly, many degenerated sequences are common to several bacterial lineages (e.g., CTAG in *cdsA* of *S. typhimurium* strain D23580 is CTGG in all other *Salmonella* strains compared here including those of *S. bongori*). Among the degenerated forms, CTGG is the most common degenerated sequence across all bacterial lineages compared throughout the genome, and many other degenerated forms, such as CTCG, are also conserved in certain locations, both reflecting a tendency of preferred nucleotide composition in the amelioration process. We anticipated that comparison of similar and dissimilar CTAG degeneration patterns among the *Salmonella* lineages may lead to novel discoveries about the divergence and evolution of the diverse pathogens originating from a common ancestor.

### Differential levels of evolutionary conservation of the CTAG sequence

We postulated that most ancestral CTAG sequences were degenerated in evolution and became unrecognizable by sequence and this postulation is at least partly supported by the results in Supplementary Table 4, in which the degeneration follows a phylogenetic trend among the bacteria. Based on this finding, we ranked the existing CTAG sequences according to their range of prevalence across bacteria of different taxonomic ranks (e.g., within a bacterial lineage, across different lineages within a genus, or between different genera) and divided them into three levels of conservation: level 1, conserved in *Salmonella*, as represented by *Salmonella* subgroup I and V strains, and *E. coli*, represented by strain K12 MG1655 (Table 4); level 2, conserved among *Salmonella* but not with *E. coli*; and level 3, conserved among only *Salmonella* subgroup I strains; genomic locations of CTAG conserved at the three levels are summarized in Supplementary Table 5.

Hypothesizing that the most highly conserved sequences may retain biological functions across the bacteria that contain them, we focused on the level 1 CTAG sequences in the least annotated intergenic regions of *Salmonella* in comparison with *E. coli*. Although intergenic sequences may be involved in replicating the genome, coordinating the expression of other functionalities, mediating recombination^24^, etc., most intergenic sequences are known for little more than insulating the genes. Since the CTAG profiles of different bacterial lineages, such as *S. typhi* vs *S. typhimurium*, may reflect differential selection pressures on specific nucleotides as suggested by phylogenetic analyses described above, we focused on the CTAG sequences in representative *Salmonella* lineages and *E. coli* as well as some more distantly related bacteria.

**Figure 3:**
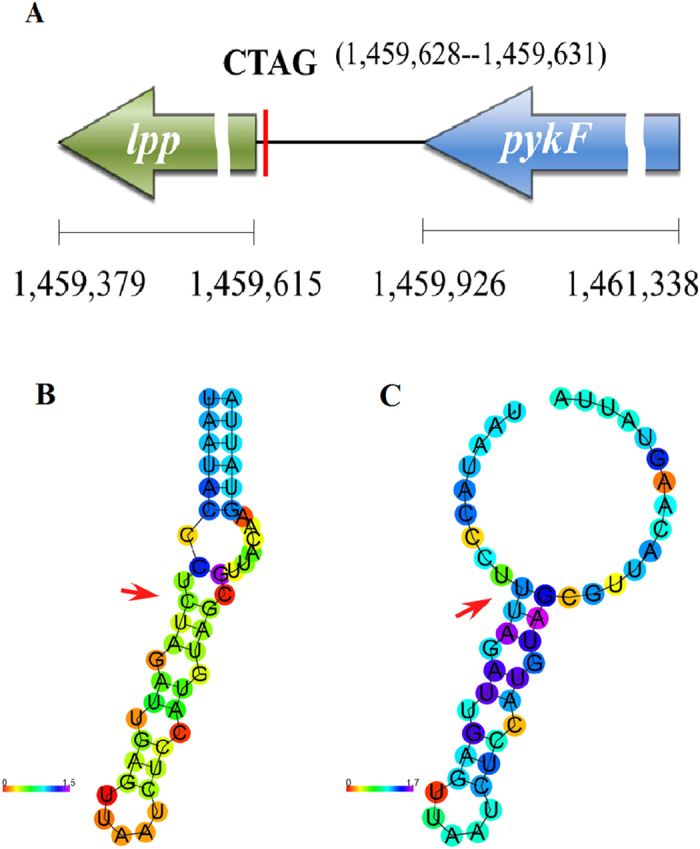
Genomic location and computer modeling of inter-lpp-pykF. (**A**) Location of *lpp*, the intergenic region and *pykF*, with the umbers at the bottom indicating the start and end nucleotides of genes *lpp* and *pykF* and the red vertical line indicating the location of CTAG (start and end nucleotides in the brackets); (**B**) Predicted stem-loop structure; (**C**) Changed structure when C in the CT(U)AG sequence is substituted by U. The positional entropy is coded by hues ranging from red (low entropy, well-defined) via green to blue and violet (high entropy, ill-defined) Predicted.

One of the intergenic regions, inter-lpp-pykF between genes *lpp* and *pykF* (Fig. 3A), is conserved among bacteria of *Salmonella,* the *E. coli* complex (including *E. coli* and *Shigella), Enterobacter, Klebsiella, Yersinia, Citrobacter, Cronobacter, Edwardsiella, Erwinia, Pantoea, Pectobacterium, Rahnella, Serratia, Shimwellia, Sodalis, Dickeya*, etc (Supplementary Table 6). The high level of conservation of this intergenic region suggests that this segment might be functionally important and may form some conformational structure for a certain biological function.

### Structure prediction of the highly conserved CTAG-containing intergenic sequences

We conducted computer modeling on inter-lpp-pykF and some other intergenic sequences that contain CTAG for further characterization using the Free Energy Minimization method^16^,^17^,^18^, which is widely used for RNA secondary structure prediction based on empirical free energy change parameters derived from experiments. This thermodynamic model assumes that an RNA molecule folds into a structure that has the minimum free energy out of the exponentially increasing possibilities with the growth of lengths of the molecule. As exemplified by the analysis of inter-lpp-pykF, we conducted the modeling and predicted a stem-loop structure using services available at http://rna.tbi.univie.ac.at/cgi-bin/RNAfold.cgi (Fig. 3B). As demonstrated by the substitution of C with U, a base change would dramatically make the free energy re-distributed among the nucleotides (Fig. 3C; compare the change of nucleotide colors) and disrupt the stem-loop structure. Note in Fig. 3C that, even if the position correlation between U and G is the same as C and G before the substitution of C by U, the structure shown on Fig. 3B (before the substitution of C by U) becomes highly unstable (Fig. 3C, after the substitution of C by U) as judged by the changed topology and the radical changes in free energy on every nucleotide. The bioinformatics prediction and biological significance need to be validated by mutational experiments.

### Continuing nucleotide refinement of the Salmonella genome to expel the CTAG tetranucleotide sequence – trend of degeneration

The differential numbers and distinct distributions of the CTAG sequence on the genomes among different *Salmonella* lineages and their degeneration patterns led us to postulate a continuing nucleotide amelioration tendency of the *Salmonella* genome to expel this tetranucleotide sequence.

We observed a common phenomenon that more-recently diverged *Salmonella* lineages tend to have greater numbers of the CTAG sequence. For example, *S. typhi*, which appeared only about 35–50 thousand years ago^25^, has 1025 CTAG sequences compared to 850 in *S. typhimurium* or 885 in *E. coli*, which have diverged millions of years prior to *S. typhi* in evolution^26^,^27^,^28^. This is mostly because of the newly acquired genomic regions, which were probably not affected by the Very Short Patch repair system before integration to the *Salmonella* genome.

**Figure 4:**
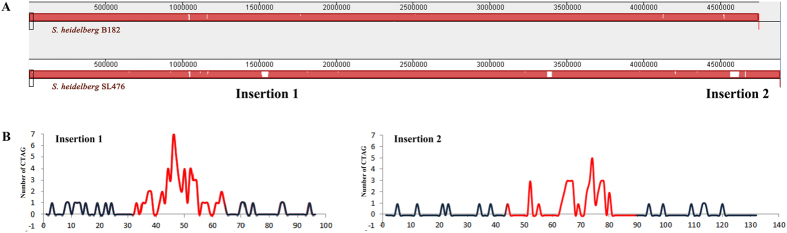
Comparison of *S. heidelberg* B182 and SL476 for their differences in CTAG profiles. (**A**) Whole genome alignment to show the two largest insertions in SL476 but not B182; (**B**) Distribution patterns of CTAG inside insertions 1 and 2. The red color indicates the regions of the insertions, with the start and end positions marked in both insertions, and black color indicates the up- and down-stream genomic sequences.

We postulated that analysis of the newly acquired genomic regions may provide a snapshot of the possible scenarios of CTAG degeneration in the *Salmonella* genome. To look into this postulation, we conducted systematic comparative analyses on two selected *S. heidelberg* strains, B182^29^ and SL476^30^, which have several large genomic insertions present in one but not in the other strain, and profiled the CTAG sequences. We first confirmed that *S. heidelberg* B182 and *S. heidelberg* SL476 belong to the same *Salmonella* natural cluster (as opposed to those of polyphyletic *Salmonella* serotypes like *S. paratyphi* B) based on their common genomic features^31^, and then compared their genomes for the numbers and distributions of the CTAG sequence. We found that for most of the genomes, the two bacterial strains have nearly identical CTAG profiles, with most of the differences being seen only in the laterally acquired DNA segments. Among the genomic insertions present only in *S. heidelberg* SL476 but not in B182 are Insertions 1 and 2 (marked Insertions 1 and 2, respectively, in Panel A and shown in red color in Panel B, Fig. 4). As anticipated, both insertions have much more densely distributed CTAG sequences than the vertically transmitted genome (the core genome). *S. heidelberg* SL476 had 108 more CTAG sequences than *S. heidelberg* B182, most of which (97 out of the 108) were located in the two largest insertions (Insertions 1 and 2; Fig. 4), where we also found many tetranucleotide sequences with one degeneracy. We are inclined to believe that many of tetranucleotide with 75% similarity to CTAG might be the degeneration products of CTAG, but this postulation remains to be validated using homologous DNA segments/sequences from diverse bacteria. The CTAG sequences within each of Insertions 1 and 2 tended to be more densely distributed toward the middle part of the insertions than the upstream- and downstream parts (Fig. 4). To see if this phenomenon might reflect a fact that *Salmonella* may tend to expel the CTAG sequences in a DNA segment by starting the process from two ends toward the center of the laterally acquired DNA segment, we profiled their CTAG sequence frequencies (Table 5). Notably, these two insertions seemed to be in different stages of the nucleotide amelioration process to degenerate the CTAG sequences since the time when they became incorporated into a *Salmonella* genome, as judged by the comparison of their calculated and profiled numbers of CTAG (Table 5). *S. heidelberg* SL476 has 833 CTAG sequences in the core genome, a number that is very similar to that of *S. heidelberg* B182 (830) and other *Salmonella* and *E. coli* strains (such as 850 in *S. typhimurium* LT2 and 885 in *E. coli* K12; see Table 1). Whereas Insertion 1 has more than eight time greater density of the CTAG sequences than the core genome, Insertion 2 has half of the density as in Insertion 1 (Table 6), suggesting that Insertion 2 has been in the genome for a much longer time than Insertion 1 to degenerate the CTAG sequences. Additionally, the greater number of CTAG sequences in Insertion 1 than in Insertion 2 is mostly in the middle part of the 41.6 kb insertion, prompting one to believe that the genomic process of degenerating the CTAG sequences had taken place inward from both terminals of the laterally acquired segment, which is illustrated in Fig. 4, where the CTAG sequences have a normal distribution in Insertion 1, but the distribution in Insertion 2 is already similar to that of the core genome. Further scrutiny of the genomic nucleotide composition refinement processes will provide novel information on bacterial genomic evolution that leads to the creation of diverse and distinct pathogens.

## Discussions

To reveal evolutionarily conserved intergenic regions for analyses of their potential functions, we previously profiled the XbaI cleavage site, which is a hexanucleotide sequence containing the tetranucleotide sequence CTAG, in representative *Salmonella* serotypes and demonstrated that the XbaI cleavage patterns are serotype-specific and so could be used to delineate *Salmonella* into natural genetic clusters^1^. Of special significance, many of the profiled XbaI cleavage sites fell in intergenic regions, indicating potential biological importance of these sequences, but their functions remain largely unknown. As profiling the XbaI cleavage site TCTAGA could sample only a subset of the sequences that contain the highly conserved CTAG tetranucleotides^2^, in this study we documented all CTAG sequences of the genome in representative *Salmonella* lineages in comparison with *E. coli*.

Overall, we found that the CTAG sequence is more than 20 times rarer than what would be estimated for a random tetranucleotide sequence consisting of one each of C, T, A and G. Most existing CTAG sequences, except those acquired relatively recently through lateral transfer, are conserved at a certain level: within a *Salmonella* serotype, among different serotypes of *Salmonella* subgroup I, across the *Salmonella* subgroups such as I and V, between *Salmonella* and *E. coli* or even more distantly related bacteria, such as CTAG in the inter-*lpp-pykF* region, which is conserved in bacteria across most genera of the Enterobacteriaceae family (See Supplementary Table 6). We believe that although many of the existing CTAG sequences in the *Salmonella* genomes may still be in the degenerating process, some of them will stay unchanged in the genomes due to their sequence importance. As such, the comparison of the CTAG degeneration patterns among different bacterial lineages should be a reasonably effective way to reveal hitherto unknown functional genomic regions according to their levels of evolutionary conservation. Computer modeling in this study supported this assumption, demonstrating that substituting any of the tetranucleotides C, T, A or G at highly conserved genomic regions like inter-lpp-pykF would disrupt the stem-loop structure (see Fig. 3).

The CTAG profiles (Table 1 and Supplementary Table 5) and their degeneration patterns (each lineage having a specific degeneration pattern; Supplementary Table 4) are unique to each of the *Salmonella* serotypes analyzed in this study as a consequence of natural selection during the adaptation of the bacteria to a given niche, e.g., a particular host. In fact, the differential levels of conservation among the existing (and very rare) CTAG sequences at different genomic locations (See Table 2 and Supplementary Table 5) should reflect their functional importance and may lead to the discovery of motifs for gene expression regulation or other biological functions. We anticipate that other bacteria may have similarly under-abundant short sequences in the genome, profiling and analysis of which may facilitate the studies of genomic evolution and biological divergence of the bacteria.

One finding of special interest in this study is that the CTAG sequences in a relatively recent insertion have a normal distribution with a typical peak in the middle of the inserted DNA segment. This phenomenon reflects a way that the genome takes to “treat” an incoming DNA segment: if not treating it as a parasite or something useless, the genome may accept it and in time modify it according to the general genomic environment. The “modification” or amelioration process may take place inward from both terminals of the horizontally acquired segment. Detailed analyses of the processes may help in understanding the biological meaning of differential codon usages in different organisms, in correlating natural selection pressure to a particular niche of the bacteria, and in uncovering novel mechanisms of genomic regulation and evolution by the recognition of highly conserved short sequences and their functions.

## Additional Information

**How to cite this article:** Tang, L. *et al*. Conserved intergenic sequences revealed by CTAG-profiling in *Salmonella*: thermodynamic modeling for function prediction. *Sci. Rep.* **7**, 43565; doi: 10.1038/srep43565 (2017).

**Publisher's note:** Springer Nature remains neutral with regard to jurisdictional claims in published maps and institutional affiliations.

## Supplementary Material

Supplementary Table 1

Supplementary Table 2

Supplementary Table 3

Supplementary Table 4

Supplementary Table 5

Supplementary Table 6

## Figures and Tables

**Table 1:** Numbers of tetranucleotide sequences consisting of one each of C, T, A and G in representative strains of *Salmonella* and *E. coli*.

	*S. typhimurium* LT2	*S. typhi* Ty2	*S. paratyphi* A ATCC9150	*S. paratyphi* B SPB7	*S. paratyphi* C RKS4594	*S. gallinarum* 287/91	*E. coli* K12
CTAG	850	1025	858	861	928	924	885
AGTC	9810	9800	9350	9985	9975	9269	9377
GACT	9693	9625	9142	9644	9686	9366	9528
GCTA	13201	12923	12504	12937	13015	12551	10608
GTAC	12993	12788	12319	13063	13053	12549	12036
TAGC	12983	13053	12516	13317	13168	12835	10606
AGCT	13948	14029	13220	14084	14016	13355	13333
TCGA	14306	13999	13511	14336	14308	13695	15457
ACGT	15426	15168	14624	15435	15423	14895	14545
TGCA	15872	15904	14995	15947	15812	15041	19761
CATG	16194	16140	15360	16326	16174	15542	15246
TACG	16501	16186	15580	16380	16402	15993	14101
CGTA	16431	16339	15759	16658	16374	15855	14324
ACTG	18472	18294	17357	18406	18318	17524	20435
CAGT	18347	18496	17303	18571	18614	17431	20477
GTCA	19434	19281	18510	19691	19871	18250	18388
GATC	19168	18787	18097	19138	18922	18455	19120
TGAC	19229	18976	17926	18981	18742	18654	18580
ATGC	21823	21318	20664	21794	21473	21041	21733
GCAT	21915	21835	20568	22005	21991	20890	21685
CTGA	24470	23934	22577	24204	24023	22834	24365
TCAG	23808	24177	22699	24459	24418	23114	24638
CGAT	26823	26068	25427	26801	26339	26146	24248
ATCG	26940	26430	25439	27020	27157	25781	24354

**Table 2:** Phylogenetic distribution of bacteria having low CTAG sequence frequencies.

Bacterial strain	Genome size (bp)	Number of CTAG	CTAG/kb	GC %
*Morganella morganii* KT	3799539	408	0.107	0.51
*Citrobacter koseri* ATCC BAA-895	4720462	609	0.129	0.54
*Enterobacter cloacae* EcWSU1	4734438	743	0.157	0.55
*Rahnella*				
*R. sp.* Y9602	4864217	810	0.167	0.52
*R. aquatilis* HX2	4962173	920	0.185	0.52
*Salmonella*				
*S. enteritidis* P125109	4685848	807	0.172	0.52
*S. typhi* CT18	4809037	1026	0.213	0.52
*S. paratyphi* A ATCC9150	4585229	858	0.187	0.52
*S. paratyphi* C RKS4594	4833080	928	0.192	0.52
*S. typhimurium* LT2	4857432	850	0.175	0.52
*S. typhimurium* DT104	4933631	957	0.194	0.52
*S. bongori* NCTC 12419	4460105	716	0.161	0.51
*Escherichia*				
*E. fergusonii* ATCC 35469	4588711	784	0.171	0.50
*E. coli* K-12 MG1655	4641652	885	0.191	0.51
*E. coli* IAI39	5132068	951	0.185	0.51
*E. coli* SE15	4717338	916	0.194	0.51
*E. coli* UM146	4993013	1034	0.207	0.51
*E. coli* O157:H7 EDL933	5528445	1176	0.213	0.50
*Shigella boydii* Sb227	4519823	1066	0.236	0.51
*Pantoea*				
*P. ananatis* LMG 5342	4605545	987	0.214	0.53
*P. sp.* At-9b	4368708	555	0.127	0.55
*Erwinia tasmaniensis* Et1/99	3883467	996	0.256	0.54

**Table 3:** Lineage-specific CTAG degeneration patterns in *Salmonella* and *E. coli* K12.

LT2 site	Gene name	*S. tm*	*S. ty*	*S. pA*	*S. pu*	*S. ga*	*S. en*	*S. ch*	*S. pC*	*S. du*	*S. he*	*S. ag*	*S. ne*	*S. sc*	*S. ja*	*S. ar*	*S. bo*	*E. coli*
257125	*pyrH&frr*	CTAG	CTAG	CTAG	CTAG	CTAG	CTAG	CTAG	CTAG	CTAG	CTAG	CTAG	CTAG	CTAG	CTAG	CTAG	CTAG	CTAC
264404	*yaeT*	CTAG	CTAG	CTAG			CTAG			CTAG	CTAG	CTAG						
440769	*yaiI&aroL*	CTAG	CTAG	CTAG	CTAG	CTAG	CTAG	CTAG	CTAG	CTAG	CTAG	CTAG	CTAG	CTAG	CTAG			
1426591	*aroH&ydiA*	CTAG	CTAG	CTAG	CTAG	CTAG	CTAG	CTAG	CTAG	CTAG	CTAG	CTAG	CTAG	CTAG	CTAG		CTAG	
1459627	*lpp&pykF*	CTAG	CTAG	CTAG	CTAG	CTAG	CTAG	CTAG	CTAG	CTAG	CTAG	CTAG	CTAG	CTAG	CTAG	CTAG	CTAG	CTAG
1597093	*marB&marA*	CTAG	CTAG	CTAG	CTAG	CTAG	CTAG	CTAG	CTAG	CTAG	CTAG	CTAG	CTAG	CTAG	CTAG		@	
1818367	*trpH&trpL*	CTAG	CTAG	CTAG	CTAG	CTAG	CTAG	CTAG	CTAG	CTAG	CTAG	CTAG	CTAG	CTAG	CTAG	CTAG	CTAG	
1818375	*trpH&trpL*	CTAG	CTAG	CTAG	CTAG	CTAG	CTAG	CTAG	CTAG	CTAG	CTAG	CTAG	CTAG	CTAG	CTAG	CTAG	CTAG	
1977607	*eda&edd*	CTAG	CTAG	CTAG	CTAG	CTAG	CTAG	CTAG	CTAG	CTAG	CTAG	CTAG	CTAG	CTAG	CTAG	CTAC	CTTG	CTTG
2023278	*yecG*	CTAG	CTAG	CTAG	CTAG	CTAG	CTAG	CTAG	CTAG	CTAG	CTAG	CTAG	CTAG	CTAG	CTAG	CTAA	CTTG	CTTG
2149464	*yeeZ&hisG*	CTAG		CTAG	CTAG	CTAG	CTAG			CTAG	CTAG	CTAG	CTAG	CTAG	CTAG	CTAG	CTAG	CTAG
2174259	*rfbI*	CTAG	CTAG	CTAG	CTAG	CTAG	CTAG			CTAG	CTAG	CTAG	CTAG	CTAG	CTAG			
2399521	*yfaX*	CTAG	CTTG	CTTG	CTTG	CTTG	CTTG	CTAG	CTAG	CTTG	CTAG	CTTG	CTAG	CTTG				
2440495	*lrhA*	CTAG	CTAT	CTAG	CTAG	CTAG	CTAG	CTAA	CTAT	CTAG	CTAA	CTAA	CTAG	CTAA	CTAT	CTAT	CTAA	
2506054	*argW&pgtE*	CTAG	CTAG	CTAG	CTAG	CTAG	CTAG	CTAG	CTAG	CTAG	CTAG	CTAG	CTAG	CTAG	CTAG			
2800018	*gltW*	CTAG	CTAG	CTAG	CTAG	CTAG	CTAG	CTAG	CTAG	CTAG	CTAG	CTAG	CTAG	CTAG	CTAG	CTAG	CTAG	CTAG
2816155	*rimM*	CTAG	CTAG	CTAG	CTAG	CTAG	CTAG	CTAG	CTAG	CTAG	CTAG	CTAG	CTAG	CTAG	CTAG	ATAG	CGAG	CGAG
2914911	*hin*	CTAG		CTAG				CTAC	CTTG		CTAG		CTAG	CTAG	CTAG			
3098680	*eno&pyrG*	CTAG	CTAG	CTAG	CTAG	CTAG	CTAG	CTAG	CTAG	CTAG	CTAG	CTAG	CTAG	CTAG	CTAG	CTAG	CTAG	CTAG
3142826	*amiC&argA*	CTAG	CTAG	CTAA	CTAG	ATAG	CTAG	CTAG	CTAG	CTAG	CTAG	CTAG	CTAG	CTAG	CTAG	CTAA		
3528054	*argR*	CTAG	CTAG	CTAG	CTAG	CTAG	CTAG	CTAG	CTAG	CTAG	CTAG	CTAG	CTAG	CTAG	CTAG	CTAG	CTAG	
3576883	*smf&def*	CTAG	CTAG	CTAG	CTAG	CTAG	CTAG	CTAG	CTAG	CTAG	CTAG	CTAG	CTAG	CTAG	CTAG	CTAG	CTAG	
3597875	*bfr&bfd*	CTAG	CTAG	CTAG	CTAG	CTAG	CTAG	CTAG	CTAG	CTAG	CTAG	CTAG	CTAG	CTAG	CTAG	CTAG	CTAG	
3834463	*yiaE*	CTAG	CTGG	CTGG		CTGG	CTGG	CTGG	CTGG	CTGG	CTAG	CTGG	CTGG	CTGG				
3910806	*rfaK&rfaZ*	CTAG	CTAG	CTAG	CTAG	CTAG	CTAG	CTAG	CTAG	CTGG	CTAG	CTAG	CTAG	CTAG	CTAG	@		
4101769	*gltU*	CTAG	CTAG	CTAG	CTAG	CTAG	CTAG	CTAG	CTAG	CTAG	CTAG	CTAG	CTAG	CTAG	CTAG	CTAG	CTAG	CTAG
4127619	*rfe*	CTAG	CTAG	CTAG	CTAG	CTAG	CTAG	@	@	CTAG	CTAG	CTAG	CTAG	CTAG	CTAA	@		
4396313	*gltV*	CTAG	CTAG	CTAG	CTAG	CTAG	CTAG	CTAG	CTAG	CTAG	CTAG	CTAG	CTAG	CTAG	CTAG	CTAG	CTAG	CTAG
4526230	*fdhF*	CTAG	@	CTGG	CTGG	CTGG	CTGG	CTGG	CTGG	CTGG	CTGG	CTGG	CTGG	CTGG	CTGG		CTGG	
4606231	*hflX*	CTAG	CTAG	CTAG	CTAG	CTAG	CTAG	CTAG	CTAG	CTAG	CTAG	CTAG	CTAG	CTAG	CTAG	CTAG	CTAG	
4856458	*yjjY&lasT*	CTAG	CTAG	CTAG	CTAG	CTAG	CTAG	CTAG	CTAG	CTAG	ATAG	CTAG	CTAG	CTAG	CTAG	CTAG	CTAG	

*S. tm: S. typhimurium* LT2; *S. ty: S. typhi* Ty2; *S. p*A*: S. paratyphi* A ATCC9150; *S. pu: S. pullorum* RKS5078; *S. ga: S. gallinarum* 287/91; *S. en: S. enteritidis* P125109; *S. ch: S. choleraesuis* B67; *S. p*C*: S. paratyphi* C RKS4594; *S. du: S. dublin* CT_02021853; *S. he: S. heidelberg* B182; *S. ag: S. agona* SL483; *S. ne: S. newport* SL254; *S. sc: S. schwarzengrund* CVM19633; *S. ja: S. javiana* CFSAN001992; *S. ar: S. arizonae* RKS2980; *S. bo: S. bongri* NCTC12419; *E. coli: E. coli* K12. Note 2: The degenerated sequences with a different nucleotide from CTAG are in *italic* and underlined. Note 3: @denotes degenerated sequence at homologous locations to CTAG in LT2 or another Salmonella genome but with two nucleotides substituted. Genomic locations of CTAG only in LT2 are given.

**Table 4:** CTAG sequences conserved between *Salmonella* and *E. coli.*

CTAG site in LT2	Gene_id	Annotation
284175	STM0242	proline tRNA synthetase
459521	STM0403 & STM0404	intergenic region between *yajB* and *queA*
500950	STM0445 & STM0446	intergenic region between *yajG* and *bolA*
1280403	STM1196 & STM1197	intergenic region between *acpP* and *fabF*
1280562	STM1197	3-oxoacyl-[acyl-carrier-protein] synthase II
1459628	STM1377 & STM1378	intergenic region between *lpp* and *pykF*
1519898	STM1444	transcriptional regulator SlyA
1794367	STM1702	RNase II
1877803	STM1780	phosphoribosylpyrophosphate synthetase
2035488	STM1943	tRNA-Cys
2496632	STM2385 & STM2386	intergenic region between *yfcB* and STM2386
2544539	STM2430 & STM2431	intergenic region between *cysK* and *ptsH*
2797006	STM2657	23 S ribosomal RNA
2797988	STM2657	23 S ribosomal RNA
2798973	STM2657	23 S ribosomal RNA
2799967	STM2658	tRNA-Sec
2800314	STM2659	16 S ribosomal RNA
2801372	STM2659	16 S ribosomal RNA
2844094	STM2692 & STM2693	intergenic region between STM2692 and STM2693
3221860	STM3060	putative cytoplasmic protein
3346112	STM3182	putative esterase
3414851	STM3245 & STM3246	intergenic region between *tdcA* and *rnpB*
3494593	STM3330	glutamate synthase, large subunit
3585835	STM3418 & STM3419	intergenic region between *rpsM* and *rpmJ*
3589847	STM3427	30 S ribosomal subunit protein S14
3593146	STM3434 & STM3435	intergenic region between *rpsC* and *rplV*
4141162	STM3933	tRNA-Leu
4631227	STM4392	primosomal replication protein N
4810992	STM4555 & STM4556	intergenic region between *leuQ* and *rsmC*

**Table 5:** Profiles of tetranucleotides consisting of one each of C, T, A and G in insertions 1 and 2 of *S. heidelberg* SL476.

	Insertion 1	Insertion 2
Calculated CTAG	162	221
CTAG	58	39
GTAC	86	126
TAGC	92	122
GACT	103	147
CGTA	111	146
TACG	112	186
AGTC	113	132
GCTA	119	130
ACGT	122	147
GTCA	141	221
TGAC	147	233
CAGT	156	301
ACTG	163	254
CATG	164	263
TCGA	168	126
ATCG	170	196
GCAT	178	289
ATGC	180	297
AGCT	189	173
TGCA	193	265
CGAT	200	219
TCAG	216	399
GATC	221	169
CTGA	224	388

**Table 6:** Profiles of the tetranucleotide CTAG in two recent insertions and the core genome of *S. heidelberg* SL476.

	Insertion 1	Insertion 2	Core Genome
Length of DNA (bp)	41606	57892	4789272
Number of profiled CTAG	58	39	833
Density of CTAG (number/kb)	1.39	0.67	0.17
Number of calculated CTAG	162	221	18648
CTAG profiled/calculated (%)	35.8	17.6	4.5
CTAG index (%)	1.6	0.785	0.212

Note: 1 Length of the core genome is the whole genome of *S. heidelberg* SL476 (4888768) minus the lengths of the two insertions; Note: 2 CTAG index is the ratio of CTAG over the total number of all 24 combinations of the tetranucleotides consisting of one each of C, T, A and G.
